# Dr. Matthias L. Lord

**Published:** 1917-01

**Authors:** 


**Dr. Matthias L. Lord,** N. Y. University 1862, died at his home in Rochester, Nov. 28, aged 77 He was a veteran of the Civil War and, from 1868 to 1885, Superintendent of the Monroe Co., N. Y., Insane Hospital.

